# Promoting childhood farm safety in Ireland

**DOI:** 10.3389/fpubh.2022.1055082

**Published:** 2022-12-12

**Authors:** John McNamara, Mohammad Mohammadrezaei, Patrick Griffin

**Affiliations:** ^1^Teagasc - Irish Agriculture and Food Development Authority, Farm Health and Safety Knowledge Transfer Unit, Kildalton, Kilkenny, Ireland; ^2^Teagasc - Irish Agriculture and Food Development Authority, Rural Economy Development Programme, Dublin, Ireland; ^3^Health and Safety Authority, The Metropolitan Building, Dublin, Ireland

**Keywords:** children, farm, injury, safety, young persons

## Introduction

Preventing injury to children and young persons related to farm work has been a priority in Ireland since the enactment of the Safety, Health and Welfare at Work Act, 1989 (SHWWA.1989) (1). Under this legislation a person in the age category of 0–18 years old or still at school are considered a child or young person and in this paper are described as a child (ren/hood). The Public Health Model of injury causation conceptualizes an injury occurrence as being due to multiple interacting physical and human factors, while the Social-Ecological Model enhances this model by defining various levels of the social and physical environment influential to persons' occupational safety and health (OSH) (2). Farms internationally are mainly small scale, with a wide range of hazards and associated risks and are dispersed throughout the countryside (3). Thus, promoting childhood safety represents a particular challenge for regulators and farm organizations. This paper describes the following related to childhood farm OSH in Ireland: (a) the socio-economic background of farming of relevance to children; (b) the legislative background; (c) trends in childhood farm injuries; (d) promotional activities with particular reference to current initiatives. The paper finishes with a discussion and conclusions section based on information presented in this paper.

## Farming in Ireland

Ireland currently has 130,216 farms with 276,000 persons employed, 73% of whom are male (4). Farming is the sole occupation of 53% of farm holders while average farm size is 32.4 hectares (80.1 acres). The average age of farm holders is 57.2 years with dairy farmers having a younger average age (52 years). Farmer age structure is skewed, with just 7.4% being aged under 35 years and 32.9% aged 65 years and over. Farm enterprise distribution varies throughout the country (5), with, beef (74.2 k), dairy (15.3 k), sheep (17.4 k) and tillage (4.6 k) along with mixed enterprises and “other” including equine, horticulture, pigs and poultry (15.0 k) being the main enterprises (4).

Farm incomes in Ireland are monitored by the National Farm Survey conducted by Teagasc (6). In 2020, average farm income was highest for dairying (€74.2 k), followed by tillage (€32.1 k), sheep (€17.9 k), beef production (€15.0 k); beef rearing (€9.1 k). Farm income varies annually but the relativities for 2020 are typical. A farm household income may be higher than farm income as in many cases off-farm employment and other transfers provide additional income.

Regarding children on farms, the National Farm Survey (7) reported their numbers for various age categories from 0 to 19 years (Table 1). Overall, 40.1% of farming families have family members in this age range with dairy farms (50.6 %) having the highest proportion followed by tillage farms (40.34%), beef production (36.7%), beef rearing (32.2%) and sheep farming (28.6%).

**Table 1:** Percentage of children/ young persons in age cohorts per enterprise on Irish farms.

	**Age category (%)**			
	**< 5**	**5–15**	**16–19**	**Total**
**Farming families (*n* = 896)**	**5.7**	**18.1**	**16.2**	**40.1**
**Enterprise (** ***n*** **, %)**				
Dairy (314, 35%)	7.0	24.5	19.1	50.6
Tillage (72, 8%)	5.6	20.8	13.9	40.3
Sheep (126, 14.1%)	2.4	11.1	15.1	28.6
Beef - Rearing (152, 17%)	5.9	14.5	11.8	32.2
Beef - Production (218, 24.3%)	5.5	1 5.1	16.1	36.7
Other (14/1.6%)	7.1	7.1	21.4	35.6

Labor input varies among enterprises with dairying having the highest workload on average. Farm Infrastructure is an important safety issue related to childhood safety. Generally farm dwellings are located adjacent to the farmyard where much of the farm work and the majority of farm workplace injuries take place. No or limited physical segregation occurs between farm dwelling and farmyard area on many farms.

Employment of migrant workers employed on Irish farms is limited (5.6%), this occurs predominantly on horticulture enterprises and larger scale dairy farms (8). Childcare is available in Irish rural areas, however specific challenges with its provision have been reported. These include low population numbers which impacts on income generation from the service and inaccessibility of training events for staff (9).

## Legislative background

Since the enactment of SHWWA 1989, the agriculture sector, including farms with self-employed holders, has been regulated and OSH has been promoted. The 1989 legislation was updated by enactment of the SHWWA 2005 (10). The principal features of the legislation, described in a previous paper (11) include: allocation of legal duties, establishment of a state agency the “Health and Safety Authority” with statutory powers to implement, promote and enforce the legislative requirements. Under Irish SHWW legislation duty holders must implement measures to protect the OSH of persons at work and also “other persons affected by work activity,” which includes children.

The legislation also provides a statutory means to enact regulations and codes of practice (COP). Regulations specify precise legal duties and requirements while a statutory COP provides authoritative guidance but permits discretion to adopt equivalent effective measures. Both specific SHWW regulations (12) and a COP (13) related to children, described in section 4 of this paper, are in place in Ireland.

SHWW (general application) regulations (14) include a requirement to report work related fatal or non-fatal injury which require medical attention or being out of work for “more than 3 days”. Reporting is also required for injuries to persons not a work, including children, who are injured due to work activity or the place of work. The implementation of these requirements, in practice, described in a previous paper (15), indicates that fatal farm workplace injury reporting works effectively in contrast to non-fatal injury reporting. Thus, data on children fatality trends is available to give a metric of progress over time.

SHWW legislation also enables statutory consultation with key stakeholders of a work sector, such as farming, and the current statutory advisory committee for the agriculture sector is known as the Farm Safety Partnership. This body has published a Farm Safety Action Plan (2021–2024) which includes goals related to OSH of children on farms (16). Producing recommendations and promotional strategies based on current research for farm children's safety, health and well-being predominate among these goals. This plan mandates stakeholder organizations to implement OSH promotional measures within their sphere of influence.

## Codes of practice related to farm childhood and young person safety

As stated, a specific statutory COP for children SHWW on farms is in place in Ireland (13). This is a legally updated COP following-on from a previous one issued after the enactment of the SHWWA 1989. This COP gives guidance on achieving OSH of children on farms. Additionally, a Farm Safety COP giving guidance on overall farm OSH including children has been in place since year 2006 (17) and has been updated in year 2017 (18). COP documents are written for adult farmers who are advised to check and follow the codes when considering work activities related to children.

The 2017 Farm Safety COP is accompanied by a Risk Assessment Document (RAD) (19). This document assists farmers to manage OSH by providing hazard assessment templates for completion for specific farm hazards (both physical and behavioral), including childhood hazards, along with an action list for controls required but not in place. Previous research reported limited identification by farmers of hazards for ‘children and older farms' (1.4% of total) for the 2006 RAD, but that childhood OSH conditions were satisfactory on 94.1% of farms assessed where children were present (36% of farms) (11). Further Irish research indicates that farmers gave little prominence to childhood safety (2).

## Fatal farm workplace injuries to children in Ireland

Data for fatal farm workplace injuries (FFWI) indicates that 10% (*n* = 21) of total occurred to children up to age 18 years for the 10-year period to 2020 (20). An occurrence has been reported of 12% (*n* = 24) and 21% (*n* = 38), respectively for the 10 years to 2015 (18) and 2005 (17). Thus it is apparent that fatal injuries to children has reduced by 45% since the 10-year period to 2005. However, as the denominator data on number of children on farms is unavailable, accurate calculation of fatality rates is not possible (21).

The most recent H.S.A data indicates that tractors, farm vehicles and machinery have been associated with most children FFWI's (85%), while drowning/asphyxiation, falling objects and electrocution each accounted for 5% of deaths (22). Half of FFWI's to children occurred to those under 7 years of age. Previously, in 2006, it was reported that tractors and machinery (58%) and drowning (21%) were associated with childhood FFWI's (17). Thus, the association between children FFWI's with farm tractors and machinery has become a more predominant factor over time.

## Promotion of childhood farm safety

Media is a powerful tool to communicate safety messages to a large and diverse farmer audience (20). Given the relative importance of the farming sector in Ireland and the close links of the population generally to farming, a vibrant farming media exists in Ireland. This media includes national TV, radio, print and social media, while messages emanate from a wide range of organizations. Media reporting includes fatal injuries reporting, H.S.A. posts and alerts and state and farming organization media releases. Media facilitates rapid and on-going transmission of messages to the farming population related to children safety.

A considerable array of educational promotional booklets and eLearning materials for children are available in Ireland. Such materials are available from both the public e.g., H.S.A (23) and private sector organizations. An example of a private venture is “Agrikids” set-up and operated by farming mother Alma Jordan, who previously worked in marketing. Agrikids provides motivating childhood safety services for the media, schools and events (24). As a further example, an Irish private sector organization, “Agri Aware,” which promotes awareness of the value of the agriculture sector, operates a “Farm Safe Schools” educational initiative (25) for primary schools for children aged 4 up to about 12/13 years. Children and young person OSH is also included in secondary school and 3rd level agricultural education syllabi.

## Discussion and conclusions

This paper outlines the measures in place in Ireland to promote childhood safety among both adult farm family members and children which are supported by statutory legal provisions. Considerable promotion of childhood safety has taken place for over 30 years and is on-going. It has been shown that children in addition to being positively influenced by OSH promotions can positively influence the attitudes of adults also (26). However, farmers conduct low levels of formal risk assessment related to childhood OSH. Numerous organizations are engaged in promoting farm childhood safety at various levels, in line with the Social-Ecological Model (2). Data available on childhood FFWI's indicates that their level has declined both in numerical terms and as a proportion of total FFWI's. However, on-going research and promotion is needed related to childhood farm OSH, to enhance progress.

## Author contributions

JM conceptualized, drafted the manuscript, and revised manuscript. PG and MM reviewed manuscript. All authors contributed to the article and approved the submitted version.
